# Quantifying the Variability in Resting-State Networks

**DOI:** 10.3390/e21090882

**Published:** 2019-09-11

**Authors:** Isaura Oliver, Jaroslav Hlinka, Jakub Kopal, Jörn Davidsen

**Affiliations:** 1 Complexity Science Group, Department of Physics and Astronomy, University of Calgary, Calgary, AB T2N 1N4, Canada; isaura.oliverialabau@ucalgary.ca; 2 Institute of Computer Science, The Czech Academy of Sciences, 117 20 Prague, Czech Republic; hlinka@cs.cas.cz (J.H.); kopalj@cs.cas.cz (J.K.); 3 National Institute of Mental Health, 250 67 Klecany, Czech Republic; 4 Department of Computing and Control Engineering, University of Chemistry and Technology, 166 28 Prague, Czech Republic; 5 Hotchkiss Brain Institute, University of Calgary, Calgary, AB T2N 4N1, Canada

**Keywords:** resting-state networks, network inference, network topology

## Abstract

Recent precision functional mapping of individual human brains has shown that individual brain organization is qualitatively different from group average estimates and that individuals exhibit distinct brain network topologies. How this variability affects the connectivity within individual resting-state networks remains an open question. This is particularly important since certain resting-state networks such as the default mode network (DMN) and the fronto-parietal network (FPN) play an important role in the early detection of neurophysiological diseases like Alzheimer’s, Parkinson’s, and attention deficit hyperactivity disorder. Using different types of similarity measures including conditional mutual information, we show here that the backbone of the functional connectivity and the direct connectivity within both the DMN and the FPN does not vary significantly between healthy individuals for the AAL brain atlas. Weaker connections do vary however, having a particularly pronounced effect on the cross-connections between DMN and FPN. Our findings suggest that the link topology of single resting-state networks is quite robust if a fixed brain atlas is used and the recordings are sufficiently long—even if the whole brain network topology between different individuals is variable.

## 1. Introduction

Understanding the principles and mechanisms underlying complex brain function and cognition is one of the major challenges of our time [1,2]. The field of network neuroscience aims to tackle this challenge by an intuitively appealing framework that allows one to map observations to a mathematical description rooted in graph theory [3]. On the level of the whole brain, functional Magnetic Resonance Imaging (fMRI) is often used as a specific way to measure the neural activity through changes in the Blood Oxygen Level Dependent (BOLD) signal [4]. Specifically, a Resting-state functional Magnetic Resonance Imaging (Rs-fMRI) is obtained when the subject is in an awake state but not doing any task. The data obtained through Rs-fMRI are then typically used to estimate the interactions between different brain regions and a network representation is derived—different brain regions correspond to nodes in a graph and links between them represent interactions [5]. Very often, these interactions are estimated using a basic linear similarity measure (Pearson’s correlation), indeed, Pearson’s correlation captures the instantaneous bivariate dependences surprisingly well [6]. However, functional connectivity (a term coined for the statistical dependence or remote neurophysiological events by Friston [7]) captures also statistical dependences due to indirect interactions, such as due to a third common driver variable. This can lead to erroneous inference or interpretation and cause bias in the estimated network properties, such as spurious small-world property of functional connectivity networks [8,9]. For the direct interactions, the term *effective connectivity* has thus been proposed [7]. Notably, brain locations can be (strictly, or in a weighted manner) clustered based on their functional connectivity patterns, giving rise to so-called resting-state networks [10]. These have been proven helpful in understanding the physiology and neurology that governs the brain [11] and they have been used as a tool for the early detection of diseases [12,13,14], including neurophysiological diseases like Alzheimer’s, Parkinson’s, and attention deficit hyperactivity disorder (ADHD). In particular, it has been found that Alzheimer’s can be detected in Rs-fMRI through changes in the default mode network (DMN) and the fronto-parietal network (FPN) [15], while Parkinson’s and ADHD mostly affect the FPN [16]. Typically, these studies are done by concatenating data over subjects in the same group because of the low resolution on fMRI data (sampling rate typically smaller than ≈1 Hz, however, due to the sluggish hemodynamic response to neuronal activity, the effective temporal resolution of fMRI signal is rather ≈0.1 Hz) and the difficulty of recording long resting-state sessions. Thus, the resting-state networks often correspond to average networks [17,18], which are robust within the same group [19] but provide less robust information on an individual subject level, unless multiple recordings for a given subject are available.

More recently, it has been realized that the variability in the functional networks between individual subjects in the same “group” (for example, the control group) needs to be taken into account [20]. In particular, while short recordings were insufficient [21], precision functional mapping of individual human brains involving much longer recordings has shown that individual brain organization is qualitatively different from group average estimates and that individuals exhibit distinct brain network topologies [22]. However, how this variability affects the connectivity within single resting-state networks if a fixed brain atlas is used has not been addressed yet sufficiently. This question is particularly relevant in the context of early neurophysiological disease detection mentioned above, where specific resting-state networks play a crucial role.

In this paper, we aim to address the question of how large the variability in the network links of the DMN and the FPN across different subjects is for the commonly used AAL brain atlas [23]. Using the same data as in [22], we focus on three different similarity measures: The common Pearson’s correlation coefficient [17,18,24], which only allows one to measure linear correlations between any two regions; partial correlations [25,26,27], which also measure linear correlations between any two regions but eliminate indirect correlations due to other regions; and Maximum Entropy Conditional Mutual Information (MECMI) [28,29], which also eliminates indirect effects due to other regions but takes into account nonlinear effects as well. We find that a well-defined backbone exists for both the FPN and the DMN that is largely independent of the chosen similarity measure and robust across the set of nine subjects. Weaker connections between brain regions do vary significantly, which affects in particular cross-connections between DMN and FPN.

## 2. Materials and Methods

### 2.1. Data

The Rs-fMRI data analyzed in this paper are from the open data set published in [22]. The set consists of the Rs-fMRI scans of ten healthy subjects, five female and five male, with an average age of 29.1±3.3. One subject was removed from the analysis as it was reported in [22] that this subject suffered from drowsiness during the sessions. Imaging for each subject was performed on a Siemens TRIO 3T MRI scanner with a Siemens 12 channel Head Matrix Coil. Each volunteer underwent MRI scanning that included 10 resting state fMRI recordings (30 min each, 5 h total), four T1-weighted images and four T2-weighted images. Functional images (TR/TE = 2200/27 ms) comprised of axial slices acquired continuously in ascending order covering the entire brain (voxel size = 4×4×4 mm${}^{3}$, matrix size = 64×64×36 voxels). A three-dimensional high-resolution T1-weighted image (TR/TE/TI = 2300/3.7/1000 ms, voxel size = 0.8×0.8×0.8 mm${}^{3}$) was used for anatomical reference. Acquired T2-weighted images were not used in the current study.

To quantify the whole-brain pattern of functional connectivity, for each subject we computed the Pearson’s correlation matrix among the regional BOLD signal time series from 90 regions defined by the Automated Anatomical Labeling (AAL) atlas [30]. This step was preceded by a standard preprocessing routine. In particular, the preprocessing was carried out using the Matlab-based CONN toolbox for the analysis of functional connectivity in resting-state or task-based fMRI data [31]. The toolbox uses standard SPM (Wellcome Department of ImagingNeuroscience, London, UK; www.fil.ion.ucl.ac.uk/spm) modules for data pre-preprocessing. The preprocessing pipeline consisted of correction of head-motion by realignment of all functional images to mean functional image, coregistration of anatomical image and mean functional image, and segmentation of anatomical image in order to create subject-specific white-matter and cerebrospinal fluid (CSF) masks. Resulting images were spatially normalized to a standard stereotaxic MNI space (Montreal Neurological Institute, MNI) with a voxel size of 2×2×2 mm${}^{3}$. The denoising steps included regression of six head-motion parameters (acquired while performing the correction of head-motion) with their first-order temporal derivatives and five principal components of white-matter and cerebrospinal fluid. The CONN toolbox implements a component-based noise correction method (CompCor) that by default estimates the noise signal by performing PCA dimensionality reduction on white-matter and cerebrospinal fluid time-series derived from particular regions [32]. The CompCor method uses noise regions of interest (ROIs) acquired while segmenting each subject’s high-resolution anatomical images [33]. Time series’ from defined regions of interest were additionally linearly detrended in order to remove possible signal drift and finally filtered by a band-pass filter with cutoff frequencies 0.004–0.1 Hz.

Due to their general importance, in this paper, we focus predominantly on the Default Mode Network (DMN) and the Fronto-Parietal Network (FPN). In the AAL brain atlas, both of them are represented by 10 regions of interest (ROIs), see Table 1. Note that two ROIs (Angular Gyrus L/R) are shared between both networks. Thus, when we analyze both DMN and FPN together (denoted as DMN+FPN in the following) we have 18 unique ROIs.

In the following, we typically consider three different cases: (i) The group case, where all recordings over all patients are concatenated; (ii) the subject case, where all recordings of a single patient are concatenated; (iii) the recording case, where individual recordings are considered (In the case of concatenated data sets, we subtracted the respective mean of each ROI before concatenating.).

### 2.2. Methodology

As mentioned above, to infer functional networks and direct networks we use three different similarity measures: Pearson’s correlation coefficient, partial correlation, and maximum entropy conditional mutual information (MECMI). The latter two measures both eliminate indirect effects due to other regions. In other words, they account for network effects in the sense that the functional association between two ROIs (a putative direct link) may result from other ROIs in the network with which these ROIs interact (i.e., resulting from indirect links).

Pearson’s correlation coefficient (CC) between two time series, $X_{k}$ and $X_{l}$, corresponding to two ROIs is given by

(1) $$\rho \left(X_{k},X_{l}\right)=\frac{1}{N-1}\frac{\sum_{i=1}^{N}\left(X_{k,i}-\mu_{X_{k}}\right)\left(X_{l,i}-\mu_{X_{l}}\right)}{\sigma_{X_{K}}\sigma_{X_{l}}},$$

where *N* is the length of the time series, $\mu_{X_{k}}$ represents the mean value of the time series $X_{k}$, and $\sigma_{X_{k}}$ is its standard deviation. Once one has calculated the Pearson correlation coefficient for all ROIs of interest, one can assemble them in the form of the correlation matrix $C_{kl}=\rho \left(X_{k},X_{l}\right)$. Significant values in the correlation matrix indicate the existence of a link between the corresponding ROIs. To establish significance here, we generated 1000 multivariate Fourier transform surrogate series [34] for each ROI and determined the 95% significance-level for each individual entry in the correlation matrix. Thresholding the correlation matrix using this criterion gives rise to the weighted functional network.

Partial correlations (PC) can be defined with the help of the inverse of the correlation matrix $C_{kl}^{-1}$ [26,35],

(2) $$\rho_{kl}=\frac{C_{kl}^{-1}}{\sqrt{C_{kk}^{-1}C_{ll}^{-1}}}.$$

The significance is established analogously to the case of the Pearson correlation coefficient described above (Note that if we use the analytical *p*-values for the partial correlation based on the assumption of Gaussian processes to establish significance instead, we obtain very similar results, albeit at a slightly smaller significance level.). The thresholded partial correlation matrix corresponds to the weighted direct connectivity matrix.

The MECMI method assigns to each pair of variables, $X_{k}$ and $X_{l}$, a connectivity strength corresponding to the conditional mutual information between these variables (conditioned on all remaining $X_{m}$) in a distribution with maximum entropy consistent with the observed univariate entropies of all variables and the observed (unconditional) mutual information of all pairs $X_{k}$ and $X_{l}$ [28].

One can easily visualize this approach, since the set theoretic formulation of information theory allows us to map information-theoretic quantities to the regions of an information diagram, a variation of a Venn diagram. The information diagram for three variables is shown in Figure 2 with the associated information-theoretic quantities labeled entropy, H(X)=−∑p(x)log(p(x)); conditional entropy, H(X|Y,Z)=−∑p(x,y,z)log(p(x|y,z)); mutual information, I(X;Y)=∑p(x,y)log(p(x,y)/(p(x)p(y))); conditional mutual information, $I\left(X;Y|Z\right)=\sum p\left(x,y,z\right)\log\left(p(x;y|z)/[p(x|z)p(y|z)]\right)$; and multivariate mutual information, I(X;Y;Z)=I(X;Y)−I(X;Y|Z). To determine the maximum entropy consistent with the given univariate entropies and mutual information, $H_{m}\left({\left\{X\right\}}_{N}\right)$, we use the mutual information and univariate entropies as constraints, and draw on the structure of information diagrams. Each univariate entropy and mutual information corresponds to a region in the information diagram that can be written as a sum of a number of *atomic regions (atoms)*. The sum over all atoms is simply $H({\left\{X\right\}}_{N})$. Thus, as seen in Figure 2, we obtain constraints of the form:

(3) $$\begin{array}{cc} \mathrm{const}=I(Y;Z)= & I(Y;Z|X)+I(X;Y;Z), \end{array}$$

(4) $$\begin{array}{cc} \mathrm{const}=H(X)= & H(X|Y,Z)+I(X;Y|Z)\\ \; & +I(X;Z|Y)+I(X;Y;Z). \end{array}$$

In general, a system of N variables results in N1 univariate entropy constraints, N2 mutual information constraints, and A=∑k=1NNk=2N−1 atoms to be determined. In the simplest case of N=3 variables, we have six constraints and A=7 regions to specify, see Figure 2. This means we only have one free parameter, making the maximization process to get $H_{m}\left({\left\{X\right\}}_{N}\right)$ particularly easy in this case—in general, there are ∑k=3NNk free parameters.

Apart from the chosen constraints defined above, there are also general constraints on the values of the subregions that are necessary for providing a valid information diagram, i.e., such that there exists a probability distribution with corresponding information-theoretic quantities. A family of such constraints (so-called Shannon inequalities) can be inferred from the fundamental requirement that, for discrete variables, (conditional) entropies and mutual informations are necessarily non-negative: (A) $H(X_{i}|{\left\{X\right\}}_{N}-X_{i})\geq 0$; (B) $I(X_{i};X_{j}|{\left\{X\right\}}_{K})\geq 0$, where i≠j and ${\left\{X\right\}}_{K}\subseteq {\left\{X\right\}}_{N}-\left\{X_{i},X_{j}\right\}$. This set of equalities is minimal in the sense that no inequality is implied by any combination of the others [36]. Not so-well known, for N≥4, there are also inequalities that are not deducible from the Shannon inequalities, however, they have not yet been fully described and are thus not included in our optimization scheme. Each of the above inequalities can also be written as a sum of atoms, e.g.,

(5) $$I\left(X_{1};X_{2}|X_{3}\right)=I\left(X_{1};X_{2}|X_{3},X_{4}\right)+I\left(X_{1};X_{2};X_{4}|X_{3}\right)\geq 0.$$

With the use of the information diagram formulation, the maximization of entropy of the multivariate distribution can be thus reformulated as a standard linear programming problem of finding the values of the information atoms, given linear constrains (equalities for the observed univariate and bivariate quantities and set of Shannon inequalities), that maximize the total entropy (Note that due to the probabilistic nature of the algorithm (random seed and numerical stopping criteria of the linear optimization scheme), the value of the maximum entropy varies by an order of $10^{-9}$ between different runs. Thus, we chose from an ensemble of 10 runs the one with the highest entropy for the analysis. Note also that we symbolize each time series $X_{k}$ into three equally probable states, which is the minimum number of states to capture nonlinear behavior.).

**Figure 1:**
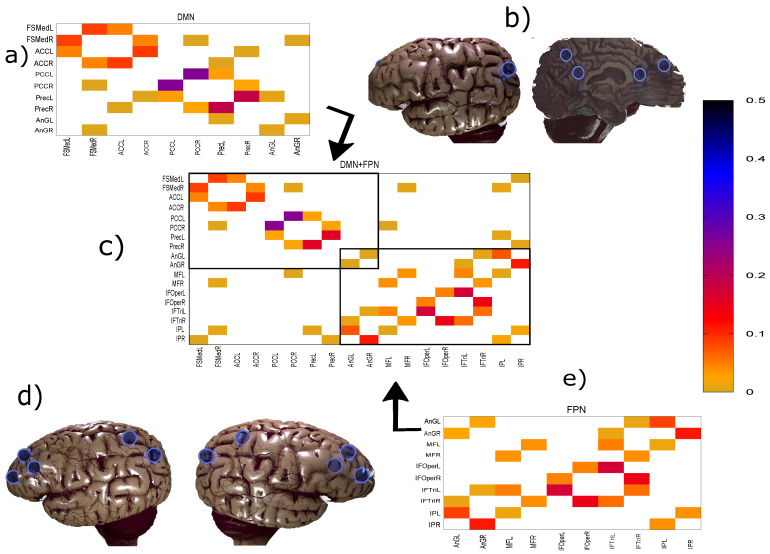
Panels (**a**), (**e**), and (**c**) show the weighted networks DMN, FPN, and both of them inferred together (DMN+FPN), respectively. Note that the differences in the inferred links in DMN only involve very weak links. The similarity measure used here is the maximum entropy conditional mutual information (MECMI), see Methodology for details. Panels (**b**) and (**d**) show the sites of the regions of interest (ROIs) in the human brain (adapted from https://www.blendswap.com/blend/13180), where (b) corresponds to DMN (only one set of the symmetrically located nodes is shown) and (d) corresponds to FPN, see Table 1 for the specific names. In panel (c), we can separate the links into two classes: Links within a network, the so-called intranetwork links, corresponding to the links inside the boxes; and cross-links between DMN and FPN, the so-called internetwork links, corresponding to the links outside the boxes.

**Figure 2:**
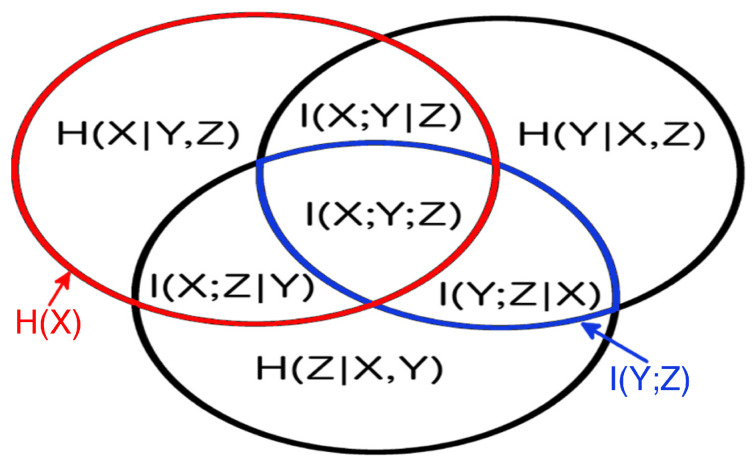
The information diagram for 3 variables. It contains 7 regions corresponding to the possible combinations of 3 variables, with their corresponding information-theoretic quantities defined in the text. The univariate entropy H(X) is the sum of all the regions in the red circle, and the mutual information I(Y;Z) is the sum of all the regions in the blue oval.

This method has proven to be efficient and able to overcome fundamental issues plaguing other estimators of conditional mutual information [29]. In particular, it does not require surrogate data to establish the significance as it can and will assign values of zero giving a trivial threshold. Thus, the conditional mutual information matrix directly gives the direct and weighted network connectivity as shown, for example, in Figure 1.

## 3. Results

### 3.1. Robustness in the Inferred Rank Order: Existence of a Well-Defined Backbone

To establish the robustness of the inferred resting-state network topology for the three considered networks (DMN, FPN and DMN+FPN), we can first look at the rank ordering: All the links between ROIs that are deemed significant (see Section 2.2) are ordered with respect to their value of the respective similarity measure.

#### 3.1.1. Group Average vs. Individual Subjects

**Figure 3:**
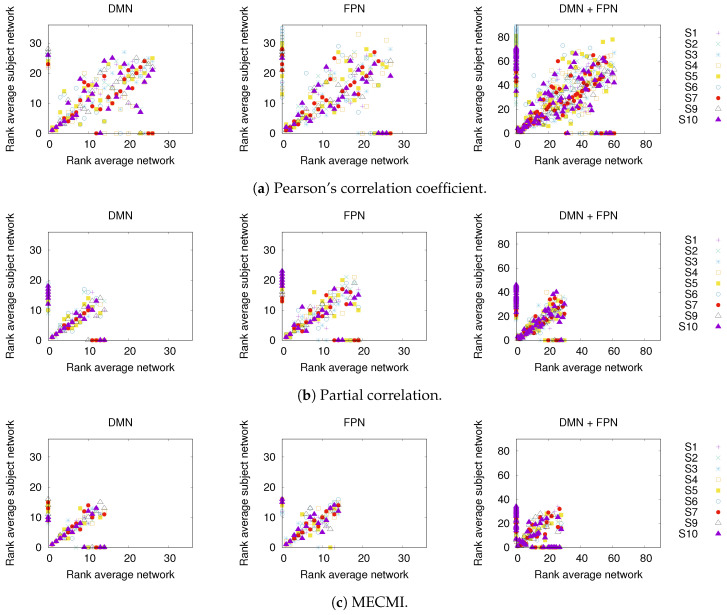
Comparison of rank ordering between group average and individual subjects (labeled S1 to S9) for the three different similarity measures, panels (**a**–**c**), and the three different networks. The rank order is by descending link strength, such that rank 1 corresponds to the strongest link. Note that a rank 0 is assigned to those links that have not been detected by a given similarity measure.

In Figure 3, a comparison between the rank ordering obtained for the group average and the nine individual subjects is shown for the different networks and the different similarity measures. Here, the rank ordering is by descending link strength. In all cases, the top ranks corresponding to the strongest links do not vary much from subject to subject. This indicates that a well-defined backbone exists, which includes about 10–20 links. In addition, the overall ranking is also quite consistent, as indicated by the linear clustering along the diagonal in Figure 3. There is also a clear tendency that the lower ranked or weaker links are more likely not to be identified.

#### 3.1.2. Robustness across Similarity Measures

**Figure 4:**
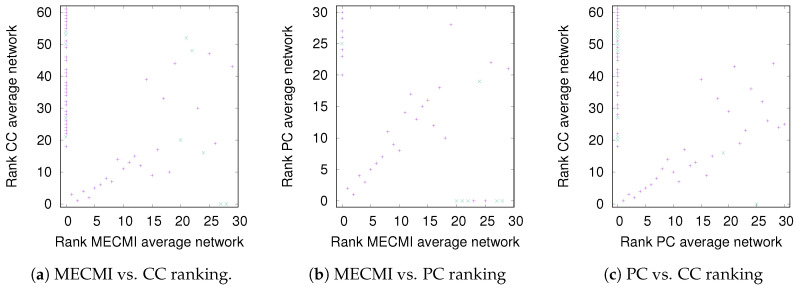
DMN+FPN: Comparison of link ranking across different similarity measures. (**a**) MECMI vs. CC; (**b**) MECMI vs. PC; (**c**) PC vs. CC. Intranetwork links are indicated by pluses and internetwork links are indicated by crosses, see Figure 1 for their definition.

While Figure 3 allows a clear comparison between group averages and individual subjects, the variations across the different similarity measures only become clear in Figure 4. Indeed, for DMN+FPN, the variation is rather small for the 15 or so highest ranked links—these strongest links are consistently identified as strongest links across all measures. In particular, the 5 strongest links are identical and include the following: IPR AnGR, PCL PCR, IFOperR IFTriR, IFOperL IFTriL, PrecL PrecR. These are also among the top 5 links if DMN and FPN are analyzed separately (Specifically, we have for DMN PrecL PrecR, PCL PCR, ACL ACR, FSMedL FSMedR, PCR PrecR, and for FPN IPR AnGR, IPL AnGL, IFOperR IFTriR, IFOperL IFTriL, IFTriL IFTriR.), see also Figure 1. The strongest links making up the backbone are almost exclusively intranetwork links. The internetwork links tend to be weaker and less numerous. In particular, there is only a single internetwork link that is consistently identified across the three similarity measures. It connects PrecL with IPL and it is ranked 16 for Pearson’s correlation coefficient, 24 for MECMI, and 19 for partial correlations. The comparison between partial correlations and the Pearson’s correlation coefficient clearly highlights that the backbone almost exclusively consists of direct links. Related to this, all links that are highly ranked using Pearson’s correlation coefficient but are not detected by either partial correlations or MECMI (rank 18, corresponding to MFR with IFOperR; rank 21, corresponding to PrecR with IPL) close a triangle, which either indicates an indirect link or a common driver in the networks constructed by the latter similarity measures.

The comparison between the two conditional/partial similarity measures, partial correlations and MECMI, in Figure 4b, shows that links that are detected by one method but not by the other one are weak. This suggests that the backbone does not contain (many) triangles since, by construction, the MECMI method almost always identifies only the two strongest links in any existing link triangles. The absence of any triangles in the backbone indicates that global network features such as clustering and, hence, small-world properties that have been detected using Pearson’s correlation coefficient and partial correlations [24,25,27] are not reflective of the backbone topology.

#### 3.1.3. Detection of the Backbone in Individual Recordings

**Figure 5:**
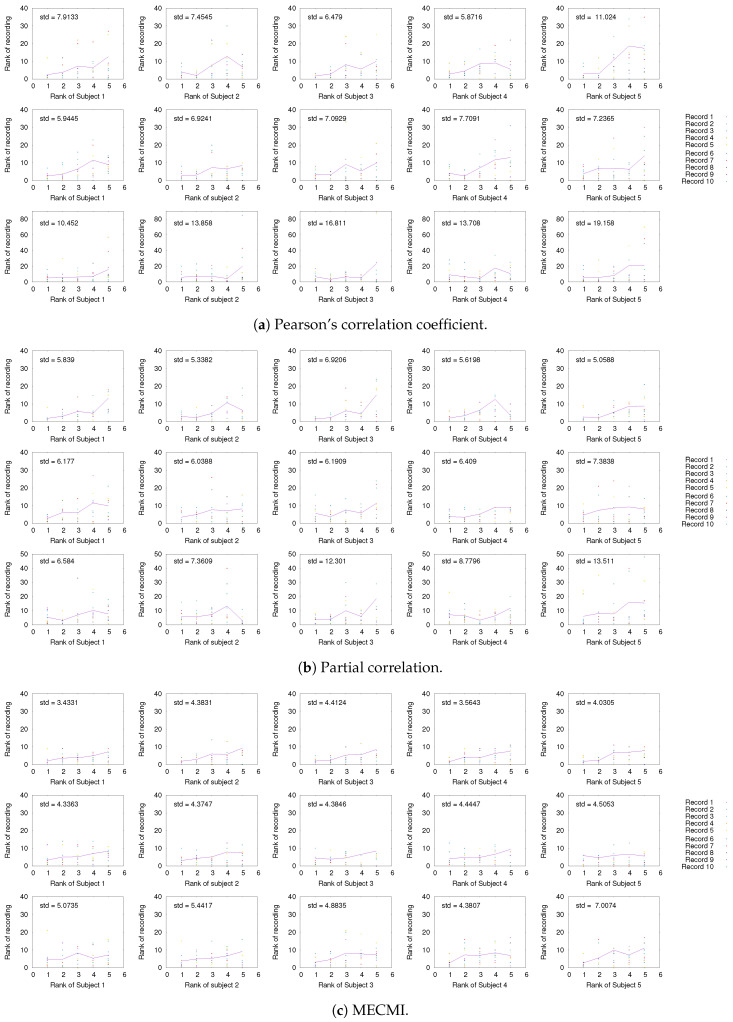
Comparison of rank ordering of the five strongest links of a given subject with those obtained for individual 30 min recordings of the same subject using (**a**) CC, (**b**) PC, and (**c**) MECMI. For each similarity measure, the top row corresponds to DMN, the middle one to FPN, and the bottom row to DMN+FPN. The solid lines give the rank averaged over the different recordings and the variation is quantified by the overall standard deviation (upper left corner). Note that if a link is not detected from a single recording (indicated by rank 0 in the panels), it is included in the average/standard deviation with a rank corresponding to the number of detected links for that recording plus one. Only subjects 1 to 5 are shown here, the other ones are shown in Figure A1.

**Figure A2:**
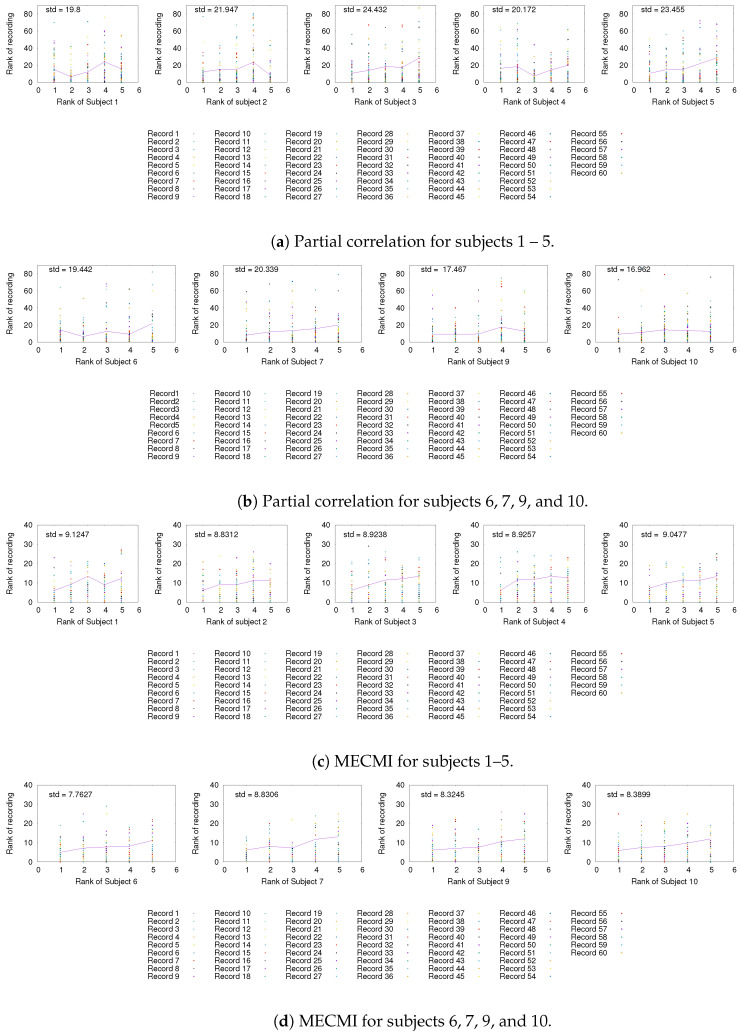
As in Figure 5 and Figure A1, but for 5 min slices instead of the full 30 min recordings.

Having established the existence of a well-defined backbone on the group and subject level, the question arises whether one can also detect it at the level of individual 30 min recordings. To address this question, we focus on the strongest 5 links (for better readability) for a given subject and look at their rank variation across the 10 individual recordings. This is shown in Figure 5. While fluctuations from recording to recording do exist, they are mostly small with a few exceptions. In particular, the backbone is consistently identified across all three networks if one uses MECMI—the mean is very close to the identity map and the variations from it are consistently lower than for the other two similarity measures. Reducing the length of the individual recordings to the more standard 5 min (see Figure A2) leads to substantial variations such that one can not reliably identify the backbone from a single recording. This is consistent with earlier studies, as discussed in the introduction.

### 3.2. Precision-Recall Analysis: Robustness of the Network Links

To quantify the robustness of the inferred network links more precisely, we can take advantage of precision-recall diagrams. If the ground truth is known, precision is defined as the ratio of correctly inferred links to all the inferred links, while recall is defined as the ratio of correctly inferred links to the number of links in the real network (see, for example, [29]). Note that the notion of “correctly inferred links” here does not take into account the inferred weights other than for determining whether the link meets the chosen significance level and it is, hence, inferred. Both precision and recall are bound to the interval [0,1] and values of 1 for both recall and precision correspond to perfect inference. Here, we use the inferred network for a single subject as our “ground truth” and compare it against the inferred network for all other subjects.

#### 3.2.1. Robustness across Similarity Measures

**Figure 6:**
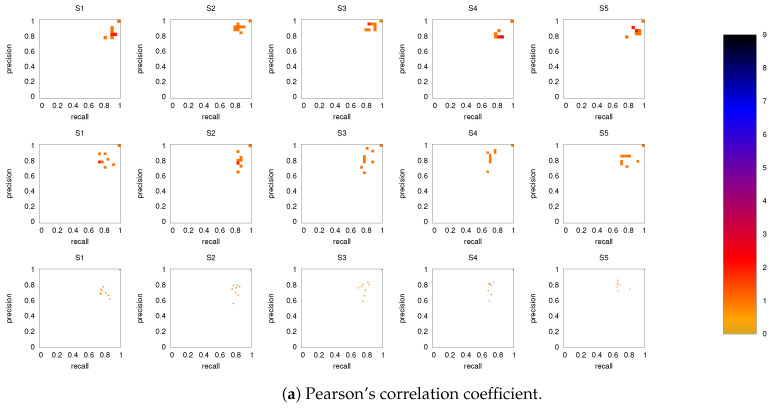

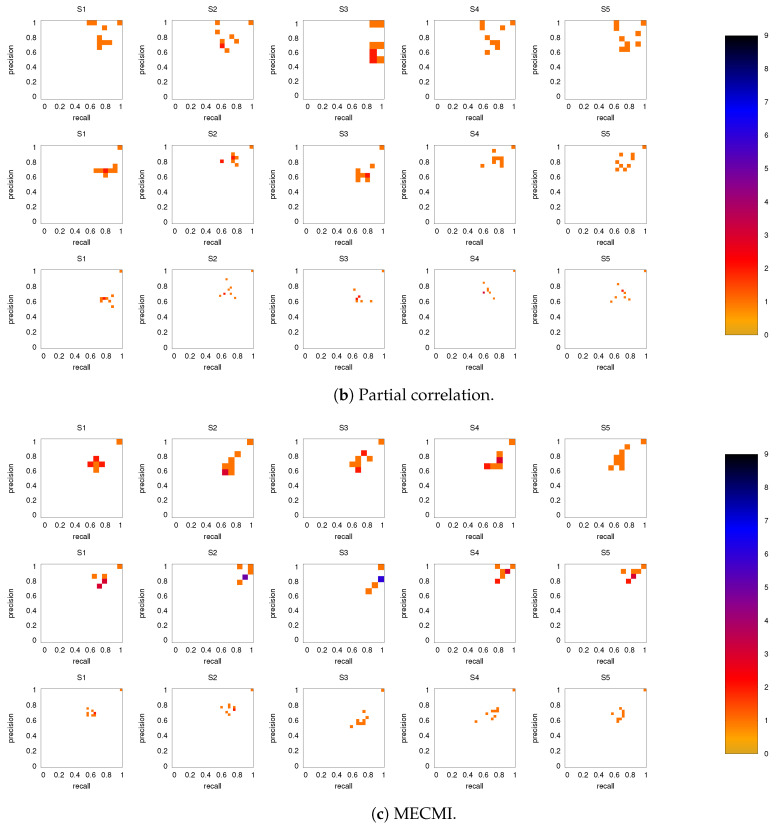
Precision-recall diagrams using the different subject networks as ground truth for (**a**) CC, (**b**) PC, and (**c**) MECMI. In all panels, the first row corresponds to DMN, the second row to FPN, and the third one to DMN+FPN. The size of the bins is ${\left(\mathrm{num}\;\mathrm{of}\;\mathrm{links}\;\mathrm{in}\;\mathrm{the}\;\mathrm{subject}\;\mathrm{network}\right)}^{-1}$. Only subjects 1 to 5 are shown here, the other ones are shown in Figure A3.

Figure 6 shows the precision-recall diagrams for all three similarity measures and all three networks. In all cases, the precision and recall values are high, confirming that most of the inferred links are consistent across subjects, as expected based on Figure 3 for both the backbone and weaker links. This is particularly true if DMN or FPN are analyzed separately.

#### 3.2.2. Robustness: Intranetwork Links vs. Internetwork Links

**Figure 7:**
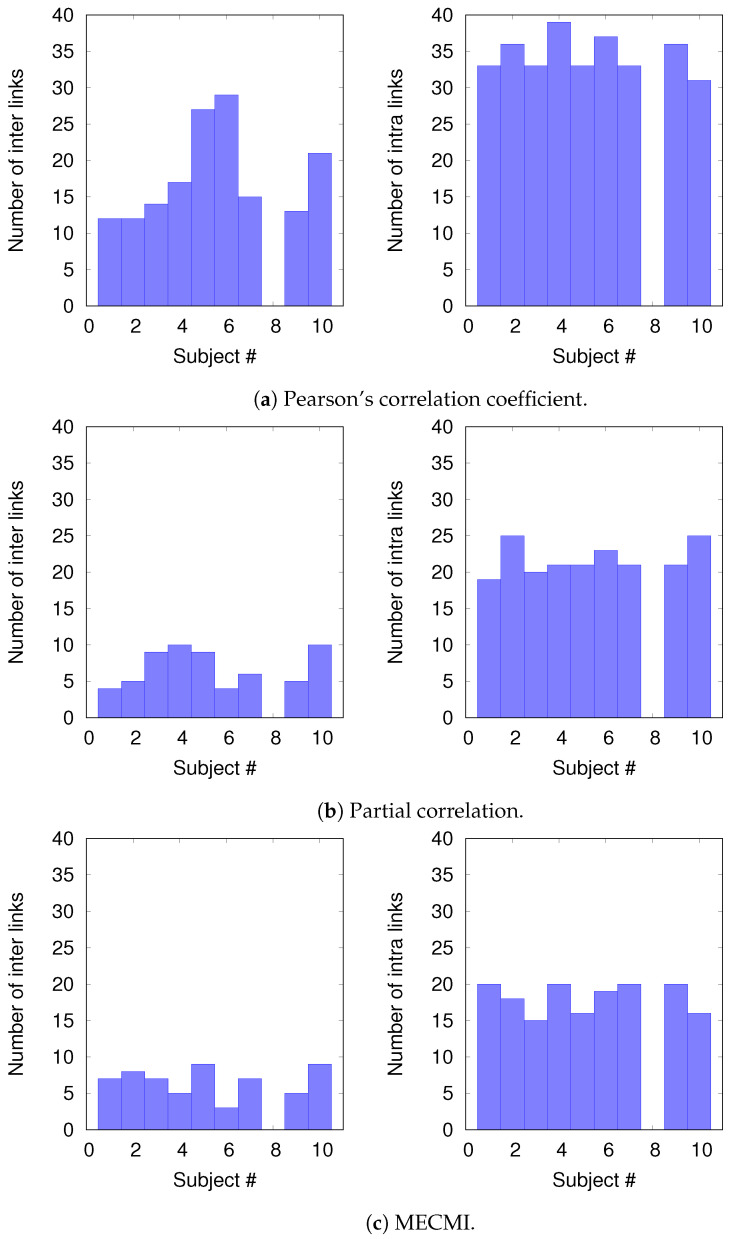
Histograms of the number of intranetwork links and internetwork links, as defined in Figure 1, for each subject and each similarity measure.

To understand the slight drop in precision and recall for the combined case DMN+FPN in Figure 6, we split the precision-recall analysis into intranetwork links and internetwork links, as defined in Figure 1. As Figure 7 shows, the number of internetwork links is typically smaller than the number of intranetwork links, as already expected based on the group averages shown in Figure 4. This difference becomes larger on average if one considers PC or MECMI instead of CC. As follows from Figure 4, the internetwork links are typically weaker than the intranetwork links.

**Figure 8:**
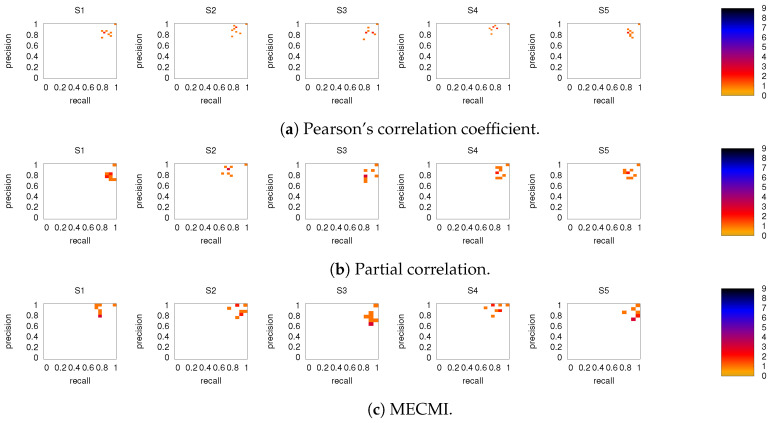
As in Figure 6, but for intranetwork links in DMN+FPN only. Only subjects 1 to 5 are shown here, the other ones are shown in Figure A4.

Figure 8 shows the precision-recall diagrams for the intranetwork links for all three similarity measures. The values are significantly higher than those for all links in DMN+FPN, shown in Figure 6, and are as high as the values for DMN and FPN (see Figure 6). This indicates that all three similarity measures perform equally well in identifying links within DMN and within FPN independent of whether the two networks are analyzed together or not.

**Figure 9:**
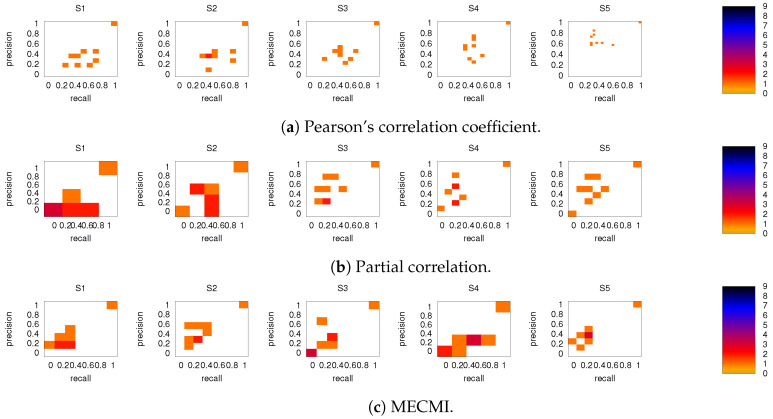
As in Figure 6, but for internetwork links in DMN+FPN only. Only subjects 1 to 5 are shown here, the other ones are shown in Figure A5.

On the other hand, the precision-recall diagrams for the internetwork links shown in Figure 9 exhibit much lower precision and recall values. This is particularly pronounced for the conditional measures. It directly explains the drop in precision and recall for the combined case DMN+FPN compared to DMN and FPN in Figure 6. Moreover, it clearly indicates that the inferred internetwork links vary significantly from subject to subject. This might be the root cause of the significant differences across subjects identified with the same recordings we use here [22].

### 3.3. Partial Analysis: Local vs. Global Conditioning

**Figure 10:**
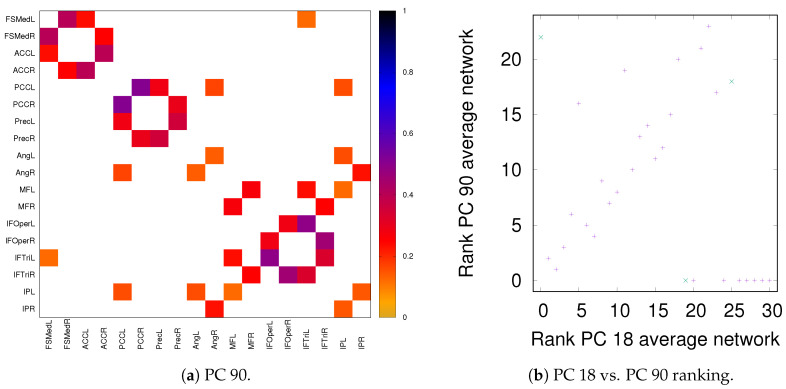
DMN+FPN: Partial correlation analysis using just the ROIs corresponding to DFM+FPN (PC 18) and all ROIs (PC 90). (**a**) Weighted network for PC 90 thresholded using the 90% significance level, analogously to Figure 1c. (**b**) Comparison of link ranking between PC 18 and PC 90—for a comparison of the weights, see Figure A6. Intranetwork links are indicated by pluses and internetwork links are indicated by crosses, as in Figure 4.

**Figure 11:**
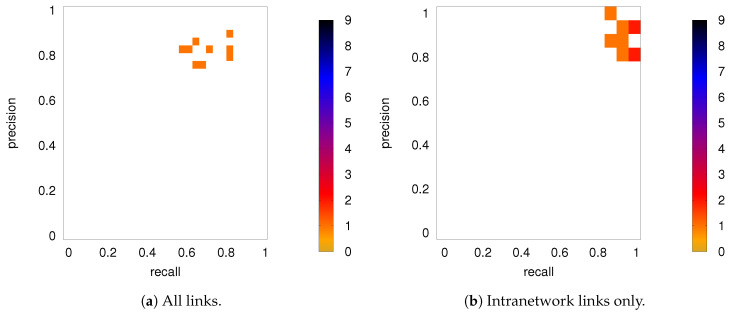
DMN+FPN: Precision-recall diagrams for the individual subjects (PC 18) while using the group average from PC 90 as ground truth, (**a**) all links and (**b**) intranetwork links only.

As discussed in Section 2.2, both partial correlation and MECMI account for network effects in the sense that the functional association between two ROIs (a putative direct link) may result from other ROIs in the network with which these ROIs interact (i.e., result from indirect links). In the previous sections, we have focused on no more than 18 ROIs (DMN+FPN) but not the full 90 ROIs. This is because in the latter case the number of ROIs is too large for i) using MECMI (due to memory and computational limitations) and ii) getting reliable estimates using partial correlations for individual recordings and also to a lesser degree for individual subjects (due to the limited amount of data). However, estimating the group average using partial correlation on all 90 ROIs does not suffer from these shortcomings and allows us to control for the influence of all ROIs, thus, eliminating spurious “direct” links that might have resulted from indirect interactions within the larger network. Figure 10a shows the weighted network (DMN+FPN) using partial correlations on all 90 ROIs. As a comparison with Figure 1c shows, the vast majority of links is identical. In particular, variations in the links only involve the weakest links. To quantify this further, Figure 10b shows a direct rank comparison between the inferred DFM+FPN networks based on partial correlations using either only those ROIs corresponding to DFM+FPN or all 90 ROIs. The sets of top 15–20 links are largely identical and only two links (rank 5 and 11 in PC 18) see a significant drop in their ranking, which indicates that some of their importance was due to indirect interactions within the larger network. Further support for the strong similarities between the two variants of partial correlation—especially with respect to the intranetwork links—comes from the precision-recall analysis shown in Figure 11.

## 4. Discussion and Conclusions

Our analysis proves the existence of a robust backbone in the considered resting-state networks, which can be reliably inferred at the group level, at the subject level, and even to a meaningful degree at the level of individual 30 min recordings. In the latter case, the limited amount of data leads to a variability between recordings for the same subject that is higher than the observed variability between subjects. All these findings are largely independent of the chosen similarity measure. Since two of the applied similarity measures are partial or conditional similarity measures, this implies that the backbone consists of direct links. The maximum entropy estimate of the conditional mutual information has the advantage that no thresholding is required to establish the significance of a link, making it a particularly powerful tool.

The relatively low variability in the direct network structure between subjects is not only present in DMN and FPN separately, similar to what has been observed in previous studies [18,19], but also when DMN and FPN are inferred together. It is crucial to realize though that an insufficient amount of data—e.g., single recordings of less than 30 min—can significantly hamper the network inference due to large statistical fluctuations [21].

Our findings shed new light on the observation that individual brain organization is qualitatively different from group average estimates and that individuals exhibit distinct brain network topologies [22]. Specifically, our findings suggest that the link topology of individual resting-state networks are relatively robust for the AAL atlas and that variations between individuals might mostly occur at the level of internetwork links. This could at least partially explain the observed differences between subjects in multiple global network measures [22], which have been thought to be largely related to the anatomically varying network pieces/ROIs across individuals. In addition, the analysis in [22] was exclusively based on using Pearson’s correlation coefficient as the similarity measure, which leads to the inclusion of weaker links that are often not direct as our study shows.

Given our findings we expect that using resting-state networks based on a fixed brain atlas as a tool for the early detection of diseases like Alzheimer’s, Parkinson’s, and ADHD should be reliable even if the brain network topology between different individuals is variable. Establishing this directly remains a challenge for the future.

## Figures and Tables

**Table 1:** Table of ROIs in the AAL brain atlas corresponding to the DMN (Default Mode Network) and the FPN (Fronto-Parietal Network). Here, L and R correspond to left (L) and right (R). See Figure 1B,D for the locations of the different ROIs.

DMN Regions	FPN Regions
Frontal Superior Medial L	Frontal Middle L
Frontal Superior Medial R	Frontal Middle R
Cingulum Anterior L	Frontal Inferior Opercularis L
Cingulum Anterior R	Frontal Inferior Opercularis R
Cingulum Posterior L	Frontal Inferior Triangular L
Cingulum Posterior R	Frontal Inferior Triangular R
Angular L	Parietal Inferior L
Angular R	Parietal Inferior R
Precuneus L	Angular L
Precuneus R	Angular R

**Figure A1:**
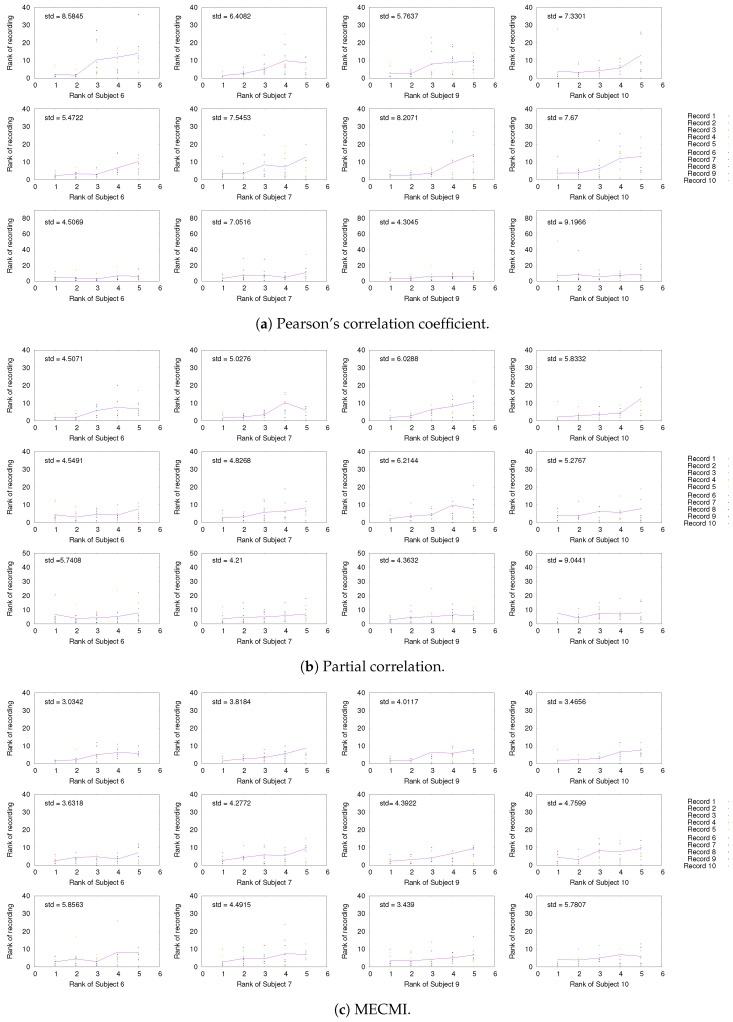
As in Figure 5, but for subjects 6, 7, 9, and 10.

**Figure A3:**
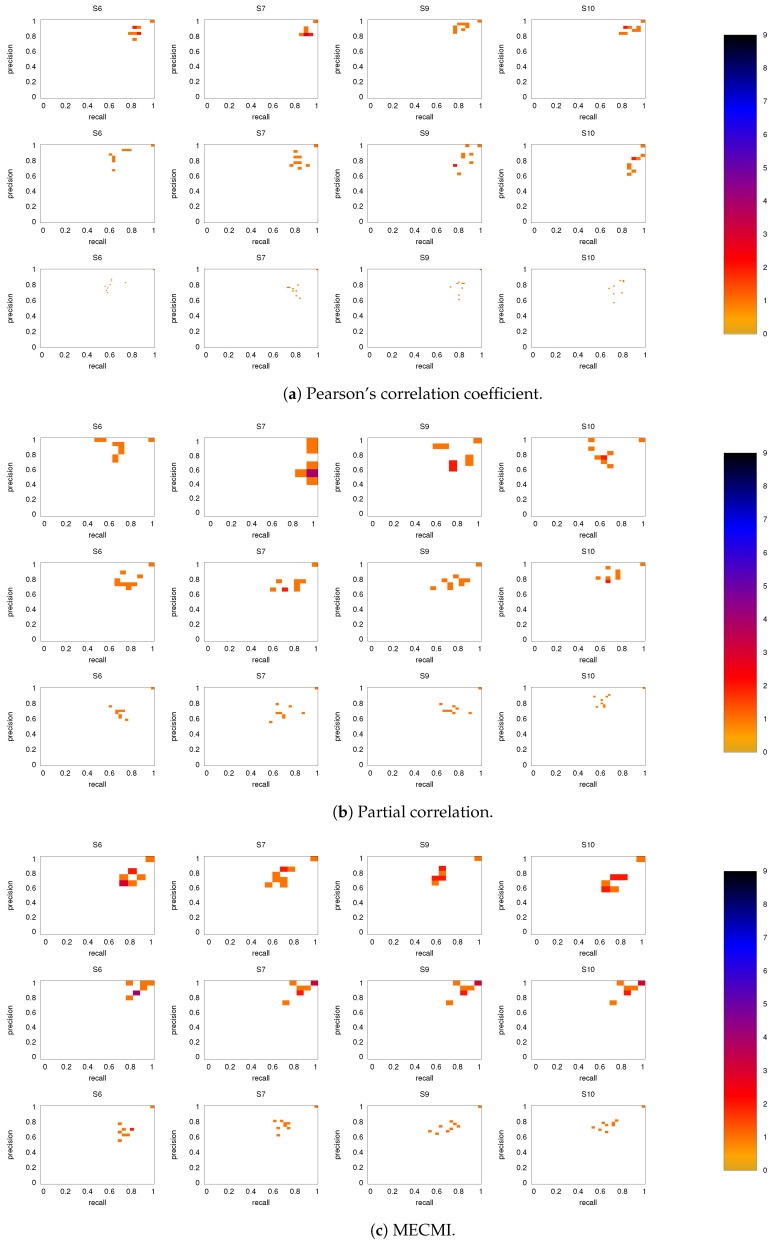
As in Figure 6, but for subjects 6, 7, 9, and 10.

**Figure A4:**
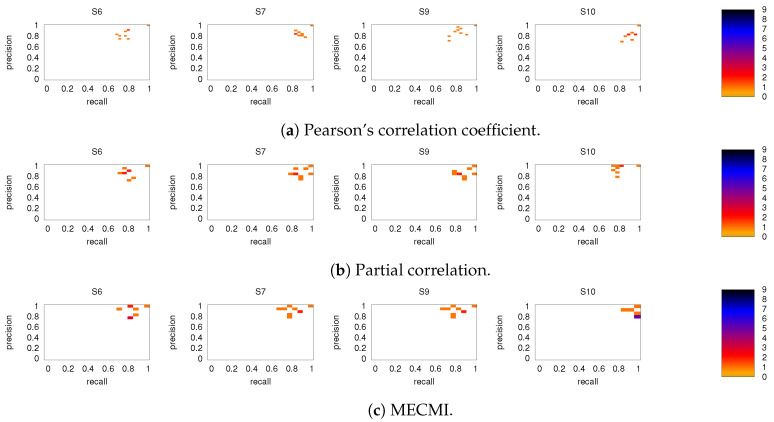
As in Figure 8, but for subjects 6, 7, 9, and 10.

**Figure A5:**
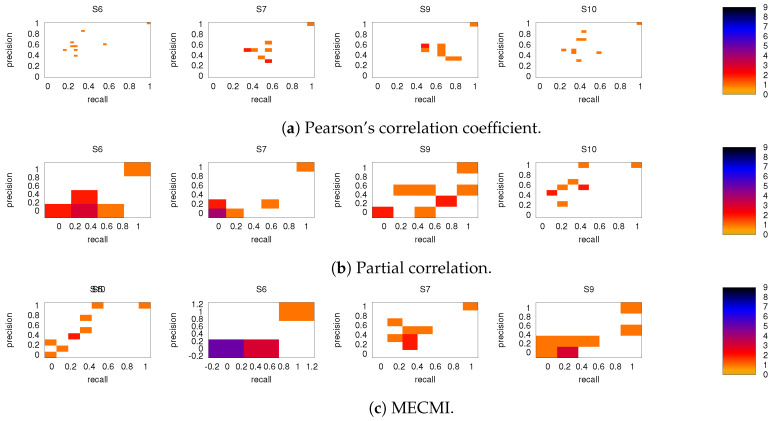
As in Figure 9, but for subjects 6, 7, 9, and 10.

**Figure A6:**
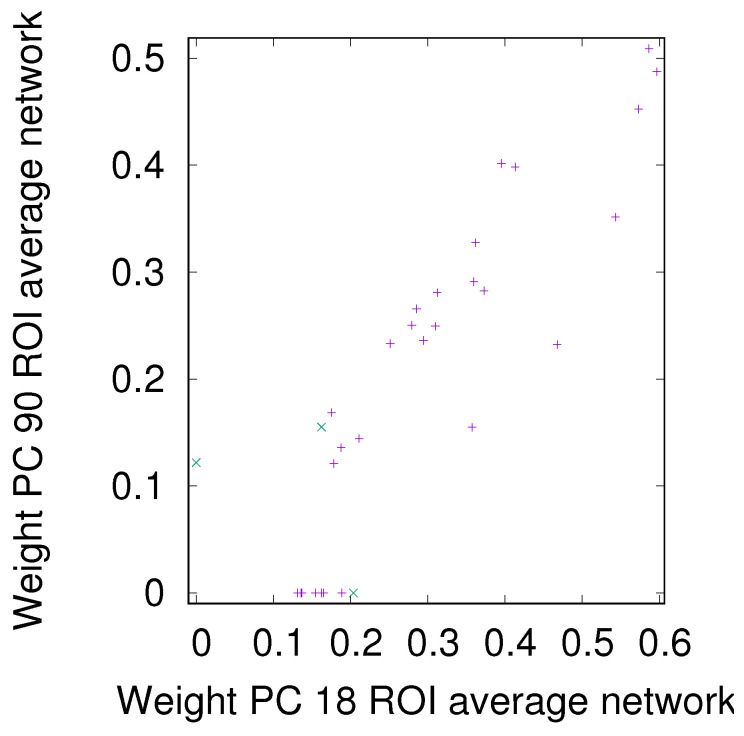
DMN+FPN: Comparison of link weights between PC 18 and PC 90, see Figure 10 for the corresponding rank comparison.
